# Supplementary material for: Identification of photoperiod-induced specific miRNAs in the adrenal glands of Sunite sheep (Ovis aries)
Source: Front Vet Sci. 2022 Jul 22;9:888207. doi: 10.3389/fvets.2022.888207 (PMC9354845; doi:10.3389/fvets.2022.888207)
Supplement: Supplementary file 1 [file Data_Sheet_1.ZIP › Supplementary file/miRDeep2 file.zip!/miRDeep2 file/Novel_Structure/SPLP42A3/Novel_251.pdf]

Provisional ID : NC_019460.2_8761
Score total : 2.2
Score for star read(s) : -1.3
Score for read counts : 0
Score for mfe : 1.9
Score for randfold : 1.6
Score for cons. seed :
Total read count : 391
Mature read count : 391
Loop read count : 0
Star read count : 0

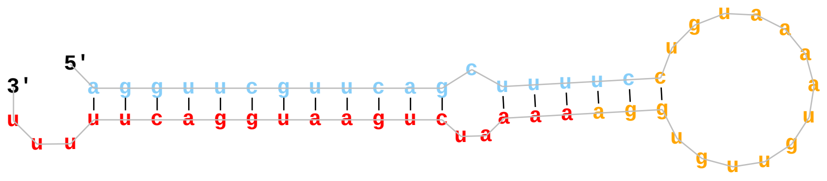

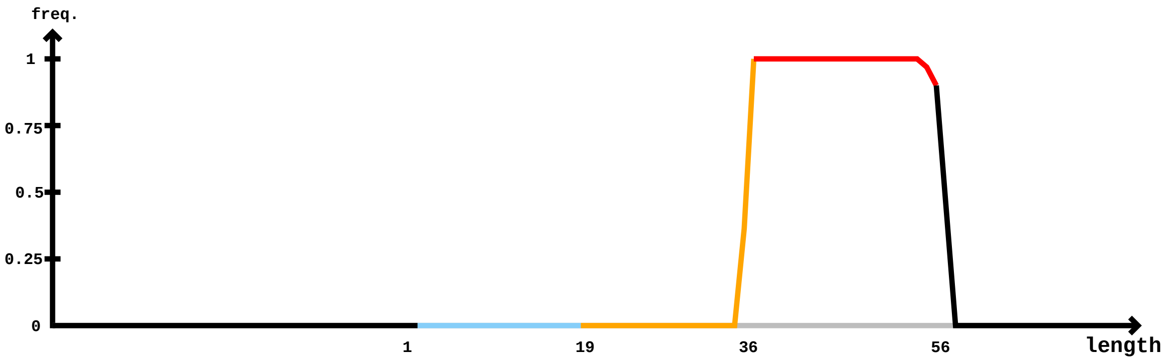


```
                                        Star                                   Mature
5'- gaaguaacugcucacagcagauacugguaggccaaagguucguucagcuuuuccuguaaaauguuguggaaaaaucugaauggacuuuuuggccaacucagcaucucccg   -3'    exp
    ..((((.(((((...))))).))))((((.((((((((((((((((((.((((((............))))))..)))))))))).)))))))).))))...........   reads     mm       sample
    .....................................................................aaaaaucugaaugAacuuu......................   12        1        seq
    .....................................................................aaaaaucugaaugAacuuuu.....................   26        1        seq
    .....................................................................aaaaaucugaaugAacuuuuu....................   78        1        seq
    .....................................................................aaaaaucugaauggacuuuuu....................   1         0        seq
    .....................................................................aaaaaucugaauggacuuuuug...................   1         0        seq
    .....................................................................aaaaaucugaaugAacuuuuug...................   24        1        seq
    ......................................................................aaaaCcugaauggacuuuu.....................   1         1        seq
    ......................................................................aaaaucugaaugAacuuuuu....................   247       1        seq
    ......................................................................aaaaucugaauggacuuuuu....................   1         0        seq
```
